# Animal Reproduction: Semen Quality Assessment, Volume II

**DOI:** 10.3390/ani15050709

**Published:** 2025-03-01

**Authors:** Anna Wysokińska

**Affiliations:** Institute of Animal Science and Fisheries, Faculty of Agricultural Sciences, University of Siedlce, 08-110 Siedlce, Poland; anna.wysokinska@uws.edu.pl; Tel.: +48-25-6431388

## 1. Introduction

Semen assessment is an important aspect of semen diagnostics used in assisted reproductive technology (ART). The most common ART technique is artificial insemination (AI), which has many advantages over natural mating. Artificial insemination technology allows the use of the best males in breeding, increases the possibility of fertilizing a larger number of females, prevents the spread of certain reproductive diseases, solves problems related to crossbreeding and mating, and enables the transport of semen over long distances [1,2]. The effectiveness of artificial insemination is closely related to the quality of the semen. Semen quality is a term that describes the probability that sperm in the ejaculate will be functional both in artificial and in natural mating [3].

Many factors affect semen quality, including semen preservation methods, type and composition of the extender, frequency of semen collection, and the individual source of the ejaculate [4,5]. As sperm quality is of key importance for the success of artificial insemination, various preservation methods are used to maintain the fertilization capacity of the sperm, such as storage of semen in liquid form and cryopreservation [6,7,8]. Sperm preservation is very important to prolong sperm survival time, increase effective semen volume, and utilize males with the best reproductive potential. Changes in sperm structure may take place during semen preservation. These changes result from the specific structure of the sperm cell membrane. The sperm cell membrane contains large amounts of polyunsaturated fatty acids (PUFA), which are susceptible to free radicals, such as ROS attacks, shortening the survival time of sperm [9,10]. The sperm cell membrane is a structure that plays a significant role in the connection of sperm with the oocyte. An intact sperm cell membrane is a condition for the proper functioning of cells and the passage of individual stages necessary for oocyte fertilization [11]. The main cause of sperm damage during cryopreservation is oxidative stress, caused primarily by elevated ROS levels [12].

Assessment of sperm function plays an important role in predicting optimal fertilization outcomes. Semen quality is influenced by numerous factors, so precise knowledge of the mechanisms influencing sperm formation is essential for accurate determination of fertilization capacity. Andrology is continually introducing new advances in the laboratory evaluation of semen. There is increasing evidence for the clinical importance of specialized tests for evaluating sperm quality. Andrological diagnostics is a continually developing area of research whose main role is to assess the reproductive potential of males. The aim of this special edition is to present the latest scientific achievements in the application of modern andrological diagnostic techniques for the evaluation of sperm quality in various animals, in order to improve their reproductive performance.

## 2. Animal Reproduction: Semen Quality Assessment—An Overview of Published Articles

The extender used for semen preservation is of key importance in maintaining optimal quality traits of sperm during storage [Zhang et al., Contribution 1]. Various components of the extender provide nutrition for the sperm, maintain the stability of the preserved environment, and prolong sperm survival time. A study using sheep of the Chinese Hu breed assessed the effect of various extenders on semen quality. Ejaculates diluted using four extenders with different components were assessed for sperm motility parameters, acrosome integrity, cell membrane integrity, and reactive oxygen species (ROS). The authors showed that sperm quality was influenced by the type of extender used. Specifically, the extender containing Tris, fructose, citric acid, and egg yolk improved the quality of Hu sheep semen during storage at 4 °C. The components of the extender were shown to be very important for maintaining optimal fertilization capacity. The effect of the composition and concentration of extender components on the quality of preserved semen has been confirmed in a study using goat semen [Li et al., Contribution 2], which showed that the glucose concentration in the extender affected sperm motility. Following incubation of the semen with various glucose concentrations, sperm motility (progressive motility and straight-line velocity), adenosine triphosphate (ATP) levels, and mitochondrial membrane potential (MMP) were shown to be significantly higher in the environment with low glucose content. The findings indicated that sperm regulate the energy metabolism pathway on the basis of changes in metabolic substrates and that low glucose levels activate oxidative phosphorylation (OXPHOS) in the mitochondria in order to maintain normal sperm functions. Low glucose conditions promote transcription and translation in mitochondria and activate mitochondrial OXPHOS to provide energy to the sperm. In addition, the LKB1/AMPK pathway is activated in order to maintain energy homeostasis and inhibit oxidative damage induced by ferroptosis. For this reason, the straight-line velocity of sperm following the use of an extender with a low glucose level is a new and important factor improving the effectiveness of artificial insemination. Hence, the dilution of semen with a low-glucose extender may be an effective and inexpensive method for improving the straight-line velocity of sperm within the female reproductive system, thus facilitating fertilization of the ovum. Another study showed that the addition of 50 mol/L FA (ferulic acid) to goat semen extender improves the quality of goat semen preserved at 17 °C [Zhang et al., Contribution 3]. The addition of 50 mol/L FA to the extender increased sperm motility, cell membrane integrity, and acrosome integrity in comparison with the control group (without the addition of FA), while at the same time reducing the peroxidation rate. These findings indicate that ferulic acid included in an extender at an appropriate concentration can mitigate oxidative damage during storage of goat semen in liquid form and improve the quality of semen preservation.

Despite the advances of recent decades, sperm damage induced by semen storage remains a common and nearly unavoidable side effect of procedures for handling and preserving semen. Cryopreservation in particular decreases sperm quality. A key role is played by the chemical cryoprotectants in the extender, which protect sperm viability in low-temperature conditions [Taheri-Khas et al., Contribution 4]. Therefore, studies are conducted to improve the quality of the cryopreservation process by perfecting the composition of extenders. An example is research conducted by Zhu et al. [Contribution 5] aimed at assessing the effect of resveratrol and its concentration on ram sperm quality following cryopreservation. The study showed that 50 μM of resveratrol added to the extender used to freeze semen can effectively mitigate the decline in sperm motility and acrosome integrity and the damage to membrane integrity taking place during cryopreservation of ram semen, while at the same time preserving the high mitochondrial activity of the sperm. Moreover, the addition of resveratrol was shown to activate phosphorylation of 5′ adenosine monophosphate-activated protein kinase (AMPK) and expression of sirtuin 1 (SIRT1), which reduces ROS production. It also enhanced the antioxidant defense system of sperm (e.g., glutathione (GSH) content and the activity of glutathione synthase (GPx), superoxide dismutase (SOD), and catalase (CAT)) and reduced apoptosis and DNA damage.

The type of extender used in semen cryopreservation affects the quality traits of sperm and in vitro fertilization rates [Pérez-Durand et al., Contribution 6]. A study using semen collected from the vas deferens of llamas (*Lama glama*) compared the effect of three semen extenders before and after cryopreservation on sperm quality parameters and rates of in vitro fertilization of llama oocytes [Pérez-Durand et al., Contribution 6]. Differences in semen characteristics following the use of the three different extenders were shown for acrosome integrity, sperm viability, membrane permeability, and sperm motility, determined before and after cryopreservation. This indicated that the extender used for cryopreservation is a factor determining rates of in vitro fertilization using sperm collected from the vas deferens of llamas. According to the authors, further research is needed to understand the effect of various extenders in order to improve rates of in vitro fertilization using sperm samples collected from the vas deferens of llamas.

Selection of the optimal method of sperm preservation is an important factor in animal breeding [Neuman et al., Contribution 7]. A study conducted on the semen of red deer analyzed the quality of sperm stored in liquid form and in the epididymides for six days at 5 °C. Sperm samples were evaluated for motility, viability, morphology, antioxidant enzyme activity (SOD, GPx and CAT), and lipid peroxidation (based on the content of malondialdehyde). Sperm stored in liquid form were shown to have greater motility and viability and better morphology and antioxidant status than sperm stored in the epididymis. The authors concluded that for short-term preservation, storage of red deer semen in liquid form is better than storage in the epididymis.

The correct dilution factor is of key importance for the survival of sperm [Zhang et al., Contribution 8]. A dilution factor that is too high or too low can reduce sperm quality and, thus, the effectiveness of semen preservation, as demonstrated for the semen of Hu sheep. Zhang et al. [Contribution 8] analyzed the effect of various dilution methods and dilution factors on sperm motility parameters and the functional integrity of sperm following cryopreservation. The authors showed that two-stage dilution (1:3 and 1:2) can improve the effectiveness of preservation of Hu sheep semen.

Semen quality is influenced by numerous genetic and environmental factors. In the case of fish breeding, temperature and hormonal stimulation play a key role [Taheri-Khas et al., Contribution 4]. The study showed that despite the administration of nearly identical doses of hormones in groups of females and males, temperature changes had differing effects on reproductive parameters. The best results in goldfish (*Carassius auratus*) breeding were obtained at 22 °C, as higher ovulation rates and better sperm quality were observed in comparison to high (28 °C) and low (16 °C) temperatures.

An important factor pointed out by Žura Žaja et al. [Contribution 9] is anthropogenic radiofrequency electromagnetic radiation (RF-EMR), which poses a potential risk to animal health, including the semen quality of boars. Two-hour exposure of the semen of breeding boars to RF-EMR at a frequency of 2500 MHz resulted in a reduction in progressive sperm motility and in the proportion of the subpopulation of sperm with a more elongated head and a larger midpiece outline. For efficient pig production and breeding, it is extremely important to determine the effect of this type of radiation on semen quality and fertilization of the sow. The authors suggest that further research is needed to investigate the impact of RF-EMR on the semen of other domesticated animal species, especially those undergoing artificial insemination procedures.

An important factor affecting reproduction in dogs is the prevalence of aerobic bacteria and mycoplasmas [Domrazek et al., Contribution 10]. By identifying and characterizing the bacteria present in dog ejaculates, we can determine potential sources of contamination and infection, which can affect semen quality. Domrazek et al. [Contribution 10] showed that *Mycoplasma* spp. is commonly present in dog ejaculates, but that semen quality is not correlated with the presence of *Mycoplasma* spp. in dogs. The authors suggest that there may be undescribed species of canine mycoplasmas that can only be identified using advanced diagnostic techniques. An understanding of the microbial composition of semen and its influence on fertility parameters is essential to the development of effective strategies for optimizing breeding outcomes in dogs. Another study using dog semen assessed the potential effects of bacteria on semen quality in dogs [Sorkyté et al., Contribution 11]. Bacteriospermia was shown to significantly affect the quality of dog semen, as indicated by the link between a higher bacterial count in the semen and reduced semen quality parameters. Not all bacteria present in the dog semen were shown to have a negative impact on its quality. Samples with *Corynebacterium* spp. were associated with a decreased bacterial load, which also resulted in better quality parameters. Other bacteria, however, were more often linked to poorer semen quality. Of particular interest in that study was the identification of β-haemolytic *Escherichia coli* as the most pathogenic bacteria for semen parameters in dogs.

Issues associated with identifying mechanisms ensuring reproduction in lizards in order to implement assisted reproductive technology were taken up by [Sánchez-Rivera et al., Contribution 12]. Given the lack of research on sperm physiology in lizards, the authors attempted to establish whether lizard sperm undergo capacitation, like mammalian sperm. The study showed that certain changes associated with sperm capacitation, such as changes in the type of movement or acrosome damage, take place after two hours of semen incubation with the capacitation medium. According to authors, there is a need to establish whether capacitation is necessary for lizard sperm to acquire fertilization competence and whether this process is crucial for improving the success of ART techniques in this group of animals.

## 3. Conclusions

This Special Issue presents several innovative studies aimed at improving semen preservation technology, various aspects of sperm analysis and preservation in different species, and also physiological processes taking place within sperm structures. The studies are very promising and provide valuable information on the biology of reproduction in mammals, reptiles, and fish. This is a continually growing area of research in which new techniques and methods are being implemented, inspiring improvements in animal reproduction. Due to the many aspects of reproduction that have yet to be explained, there is a need for further studies with the ultimate goal of maximizing the efficiency of ART.
